# Identification and validation of microglia-associated genes in ischemic stroke using single-cell and bulk RNA-seq

**DOI:** 10.1186/s13041-025-01259-x

**Published:** 2025-12-07

**Authors:** Dongliang Qian, Shuangshuang Lu, Yuanyuan Hu, Bing Leng, Xuanfeng Qin

**Affiliations:** 1 Department of Neurosurgery, National Center for Neurological Disorders, Huashan Hospital, Shanghai Medical College, Fudan University, Shanghai, 200040 China; 2 Nursing Department, Huashan Hospital, Fudan University, Shanghai, 200040 China

**Keywords:** Ischemic stroke, Microglia, Immune infiltration, Single-cell RNA sequencing, PPI Network, hdWGCNA, Pathway enrichment analysis, miRNA regulation, Drug prediction, Molecular docking

## Abstract

Ischemic stroke (IS) is an acute cerebrovascular disease characterized by high incidence and mortality. The mechanism of microglia in the pathogenesis of IS remains unclear. This study aimed to explore the key genes related to microglia in IS and their molecular mechanisms in the pathogenesis. In this study, the transcriptome data of IS were retrieved from public databases. Subsequently, candidate genes were identified through the intersection of microglia-related genes (MGGs) obtained via single-cell annotation and high-dimensional weighted gene co-expression network analysis (hdWGCNA) with differentially expressed genes (DEGs). Next, key genes were determined through protein–protein interaction (PPI) analysis and verification of expression levels. Afterwards, enrichment analysis, variation analysis, construction of regulatory networks, drug prediction, and molecular docking were performed to evaluate the role of key genes in the pathogenesis of IS. Ultimately, the quantitative real-time PCR (qRT-PCR) was applied to confirm the expression levels of DEGs in brain tissues between sham and transient middle cerebral artery occlusion (tMCAO) mice. A total of 1407 DEGs intersected with 100 MGGs, yielding 51 candidate genes. Subsequently, 3 key genes (Cd14, Csf1, and Tlr2) were successfully obtained. The study revealed that these 3 key genes were co-enriched in 4 pathways, such as leishmania infection and ribosomal, and there were differences in the enriched pathways among groups. Notably, the expression of the 3 key genes was regulated by multiple factors, including 32 microRNAs (miRNAs), such as mmu-miR-3072-5p and mmu-miR-3970, and 7 transcription factors (TFs), such as Sp1 and Nfkb1. Meanwhile, these 3 key genes predicted 8 common drugs. Interestingly, Tlr2 and Adapalene exhibited a strong binding affinity (− 9.73 kcal/mol). qRT-PCR analysis revealed significantly elevated mRNA expression levels of Cd14, Csf1, and Tlr2 in tMCAO mice compared to sham-operated controls (*p* < 0.01). This study identified and validated 3 key genes (Cd14, Csf1, and Tlr2) associated with IS, which may serve as novel targets for IS diagnosis and treatment strategies.

**Supplementary Information:**

The online version contains supplementary material available at 10.1186/s13041-025-01259-x.

## Introduction

Ischemic stroke (IS), an acute brain injury caused by interrupted cerebral blood flow, accounts for 87% of strokes and is a leading global cause of death and disability [1]. Its etiology associated with hypertension, diabetes, and atherosclerosis, while pathogenesis involves complex mechanisms including inflammation, oxidative stress, and blood–brain barrier (BBB) disruption [2]. Current reperfusion therapies, including thrombolysis and thrombectomy, are limited by narrow time windows (< 4.5 h) and hemorrhagic complications [3], while neuroprotective strategies targeting oxidative stress or glutamate excitotoxicity have demonstrated limited clinical translatability due to off-target effects or insufficient efficacy [4]. Therefore, elucidating the diagnostic criteria, etiological drivers, and pathogenic mechanisms of IS, coupled with identifying diagnostic biomarkers linked to targeted therapeutic pathways, may advance precision medicine approaches to optimize clinical management and outcomes.

Microglia, the CNS-resident immune cells, regulate neural homeostasis and neuroinflammation through dynamic polarization into pro-inflammatory (M1) or reparative (M2) phenotypes [5]. Following stroke, microglia rapidly activate in response to danger signals like ATP, glutamate, and high mobility group box 1(HMGB1), triggering pro-inflammatory responses via receptors such as purinergic (P2X, P2Y), TLRs, and inflammasomes such as NLRP3, which drive cytokine release and exacerbate neuronal damage [5]. Concurrently, they undergo metabolic shifts toward glycolysis and lipid droplet accumulation, supporting phagocytosis and proliferation but potentially leading to dysfunctional foam cells in chronic stages [6, 7]. Despite advances, the mechanistic basis through which MRGs coordinate phenotypic transitions remains incompletely defined. This knowledge gap is compounded by the paucity of integrated multi-omic datasets that could enable systematic discovery of MRG-associated therapeutic targets and stage-specific disease biomarkers.Elucidating MRGs could unveil novel strategies to modulate microglia responses, improving IS outcomes.

Single-cell RNA sequencing (scRNA-seq) has revolutionized IS research by resolving cellular heterogeneity and dynamic functional states at unprecedented resolution. This technology enables precise mapping of transcriptional profiles across diverse cell types, including microglia, neurons, and vascular endothelial cells, unveiling their roles in neuroinflammation, blood–brain barrier (BBB) disruption, and tissue repair. Recent studies employing scRNA-seq in aged murine IS models revealed dynamic shifts in brain immune cells post-stroke, identifying a potentially stroke-specific microglial subset (MG6) and functionally distinct myeloid subpopulations, providing critical insights for therapies targeting immune subsets [8]. For example, integrated spatial transcriptomics uncovered GALECTIN-mediated microglia-astrocyte crosstalk promoting recovery. Recent studies utilizing scRNA-seq have further elucidated synergistic neuroprotective mechanisms involving KBA and Z-GS in IS, identifying Spp1 as a pivotal mediator of their interaction, which may guide the development of targeted therapies against this key molecular hub [9]. However, challenges persist in translating single-cell insights into clinical applications, necessitating multi-omics integration and functional validation to bridge molecular discoveries with therapeutic innovation.

In this study, publicly available murine single-cell datasets were used to identify microglia in IS. Cell–cell communication and pseudotime trajectory analyses were employed to map their interaction networks and activation dynamics. hdWGCNA screened MGGs, which were intersected with IS DEGs. Key genes were prioritized via PPI networks and expression validation, followed by functional enrichment, molecular regulatory network analysis, drug prediction, molecular docking, and qRT-PCR validation in tMCAO and sham mouse brain tissues. This approach provides novel insights into microglia-related mechanisms for IS diagnostic and therapeutic targets.

## Results

### A total of 18 types of cells were identified in single cells

**Fig. 1:**
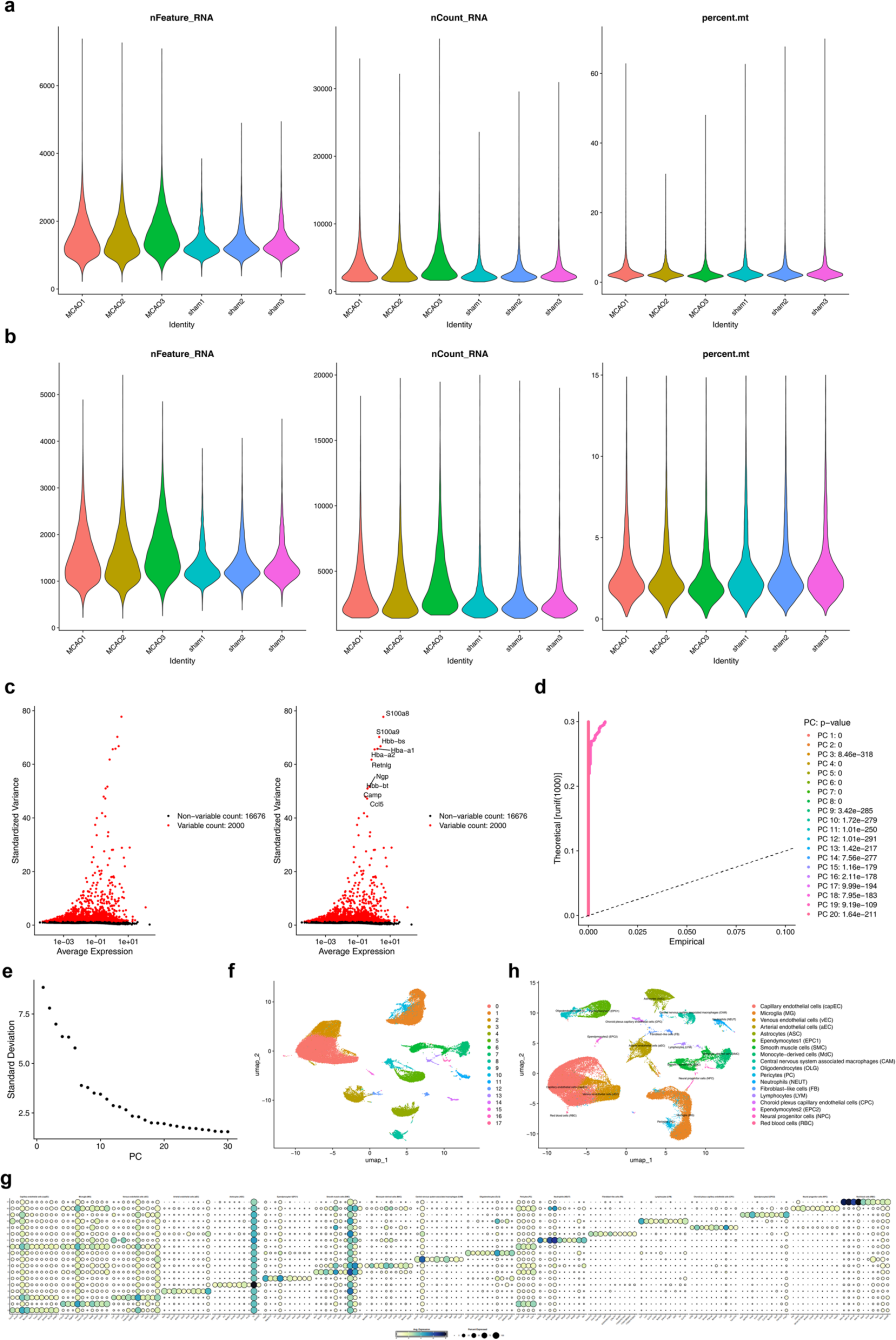
IS single-cell data preprocessing. **a** nFeature_RNA, nCount_RNA, and percent.mt plots of pre-QC dataset data, **b** nFeature_RNA, nCount_RNA, and percent.mt plots of the dataset data after QC, the abscissa is different grouping information, the ordinate is the count information, and the black dots represent individual cells. **c** Scatter plot of high variation genes, the horizontal axis represents the average expression level of the gene, the vertical axis represents the normalized variance, each dot in the graph represents a gene, and the color and shape of the dot indicate the variability of the gene, **d** PCA Principal Component Analysis Diagram, **e** Identification scatter plots for available dimensions, **f** UMAP plot of cell cluster classification. **g** Expression of Marker genes in different cell types, the abscissa represents different genes, each gene occupies a row in the graph, and the ordinate represents the different clusters obtained by dimensionality reduction, each clusters occupy a column. **h** UMAP annotation plot of cells of different cell types

First, the raw data of the single-cell dataset GSE174574 was subjected to QC (Quality Control) processing for subsequent analysis. Figure 1a, b showed the data before and after QC processing. Before processing, there were 18,676 genes and 58,523 cells. After QC, 18,676 genes and 58,025 cells were altogether selected for subsequent analysis. Second, the top 2000 HVGs were identified. The top 10 genes with the greatest variability included S100a8, S100a9, Hbb-bs, Hbb-a1, and Hba-a2. (Fig. 1c). Third, the PCA results indicated that there were no obvious outlier samples in the data. However, the significance decreased after PC = 21, and the curve in the PC scree plot tended to plateau at PC = 21. Therefore, 21 principal components were selected for further analysis (Fig. 1d, e). After that, UMAP (Uniform Manifold Approximation and Projection) clustering divided the cells into 18 cell types (Fig. 1f). On the basis of the expression intensities of marker genes (Fig. 1g), the cell clusters in the disease group and the control group were annotated as 18 cell types: Vascular smooth muscle cells (SMC), Perivascular fibroblast-like cells (FB), Central nervous system (CNS), Central associated macrophages (CAM), Monocyte-derived cells (MdC), Venous endothelial cells (vEC), Capillary endothelial cells (capEC), Arterial endothelial cells (aEC), Pericytes (PC), Choroid plexus capillary endothelial cells (CPC), Ependymocytes (EPC), Microglia (MG), Neutrophils (NEUT), Astrocytes (ASC), Lymphocytes (LYM), Oligodendrocytes (OLG), Neural progenitor cells (NPC), and Red blood cells (RBC) (Fig. 1h).

### Pseudotime analysis of microglia and cell communication analysis

**Fig. 2:**
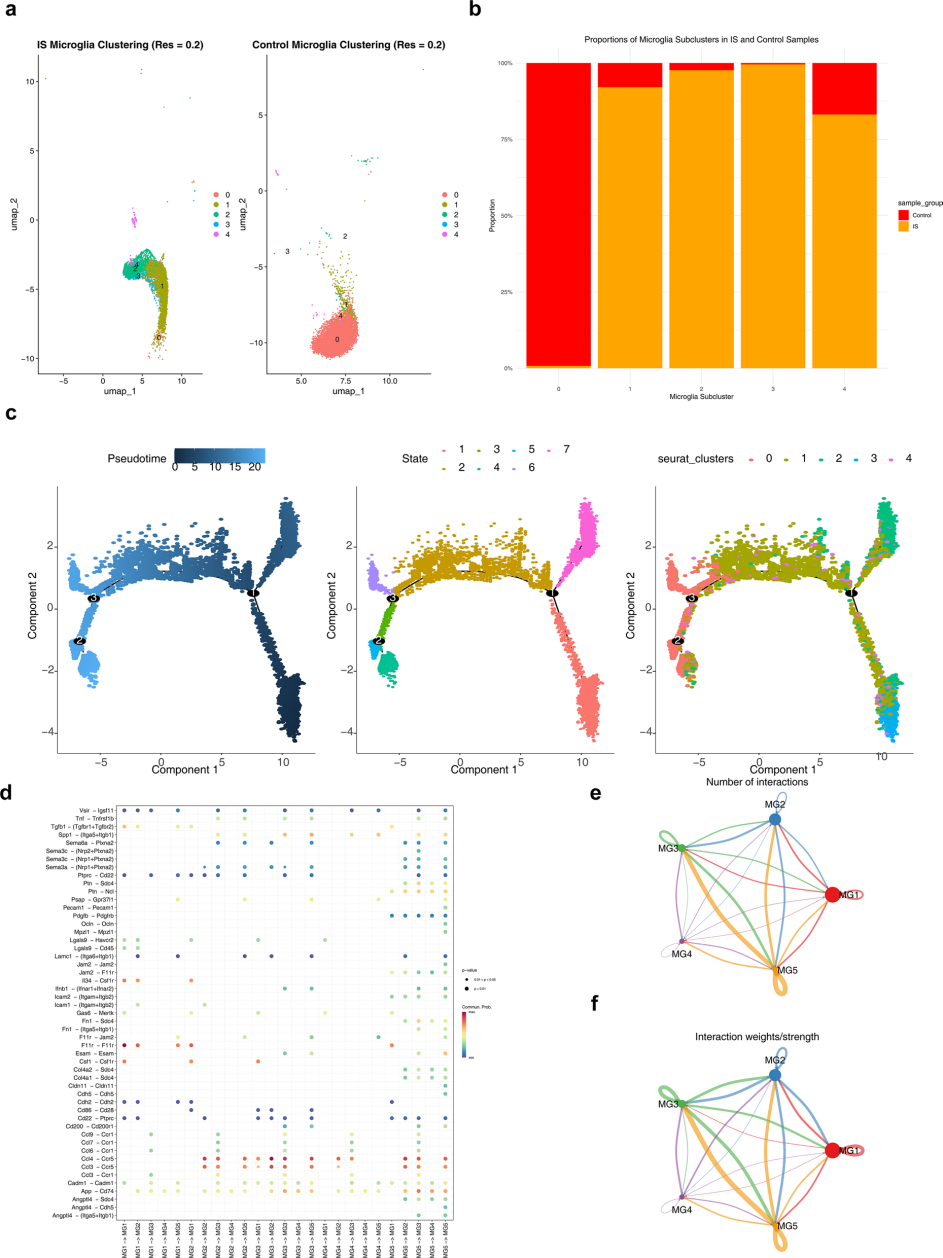
Results of the pseudo-time analysis. **a** Distribution of different subgroups of microglia in disease group and control group, **b** difference in the proportion of microglia in different samples, **c** differentiation trajectory of microglia (from 1 to 3 to cell differentiation time trajectory, cell state trajectory, cell type trajectory), **d** ligand-receptor interaction point diagram, **e**–**f** number and intensity of interactions between microglia subgroups

Prior to pseudotime analysis, microglia were subdivided into 5 subpopulations (Fig. 2a). Of the 5 subpopulations of cells, one of them accounted for the largest proportion in the control group, and the other 4 subpopulations accounted for the largest proportion in the disease group (Fig. 2b). Pseudotime analysis showed that microglia developed sequentially into different branches from their developmental starting positions, and passed through a total of 7 different developmental stages (Fig. 2c). Signal exchanges between subgroups of microglia and other cells were more frequent (Fig. 2d), and the intensity of signal exchanges between subgroups MG5 and MG3 was higher than that of other subgroups (Fig. 2e–f).

### A total of 100 MGGs were obtained

**Fig. 3:**
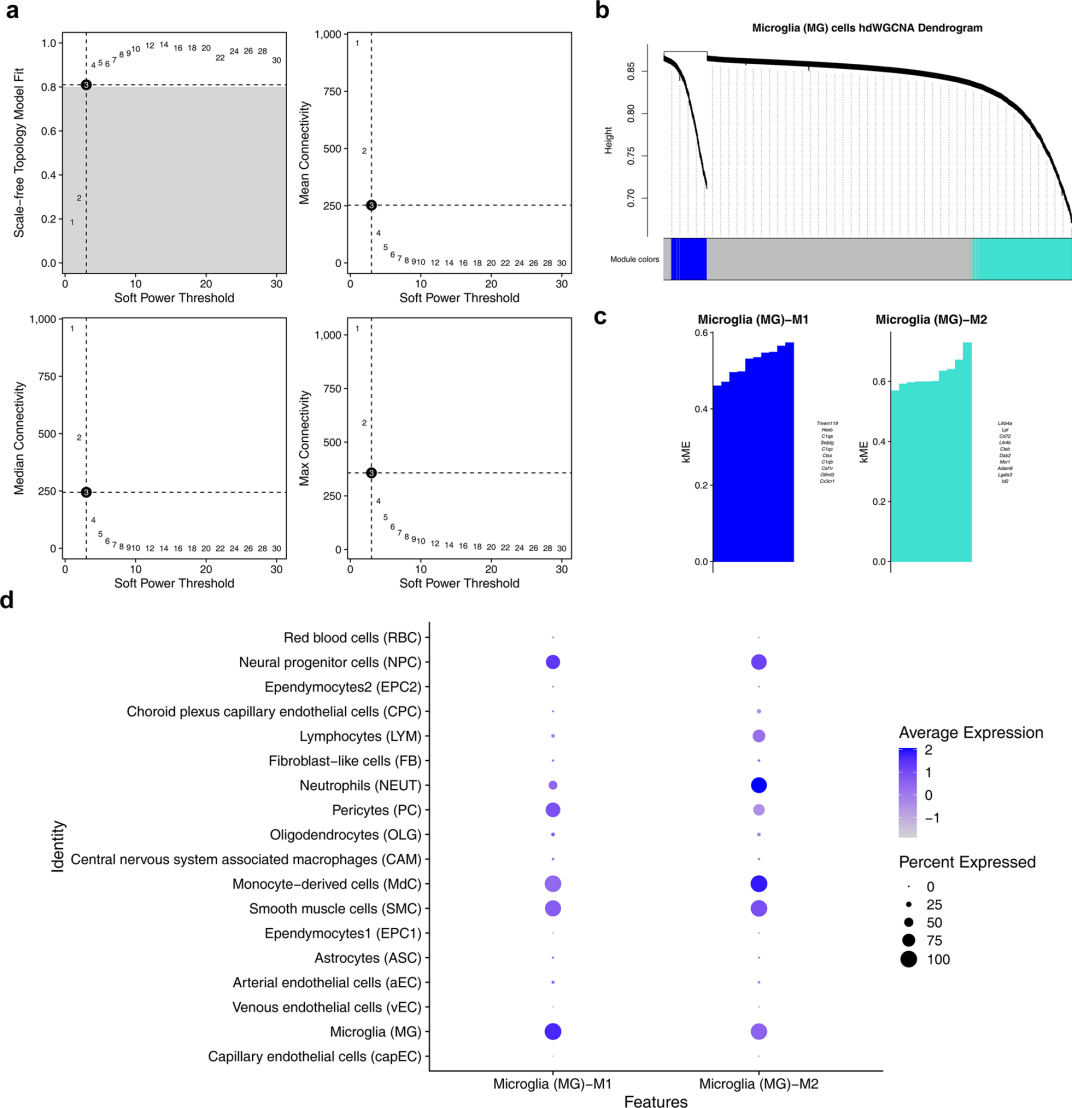
WCGNA’s analysis results. **a** Scale-free index and average connectivity plot for soft thresholds, **b** hierarchical clustering tree, **c** main candidate characteristic genes of different modules, **d** correlation between candidate trait genes in the module

When the number of module genes was configured to 50, the optimal soft threshold of 3 was selected to construct a hierarchical clustering tree (Fig. 3a, b). Subsequently, the main genes of 2 gene modules, which were significantly associated with microglia, were screened out. These 2 modules were the blue-colored module and the cyan-colored module respectively (Fig. 3c). Finally, the correlations between the 2 gene modules and the other 18 modules were obtained (Fig. 3d). The gene modules of Red blood cells (RBC) and Neural progenitor cells (NPC) both had relatively high average expression levels regarding module eigen-gene characteristics. Ultimately, 100 MGGs in the 2 gene modules were obtained.

### Functional enrichment and age association analysis of 51 candidate genes

**Fig. 4:**
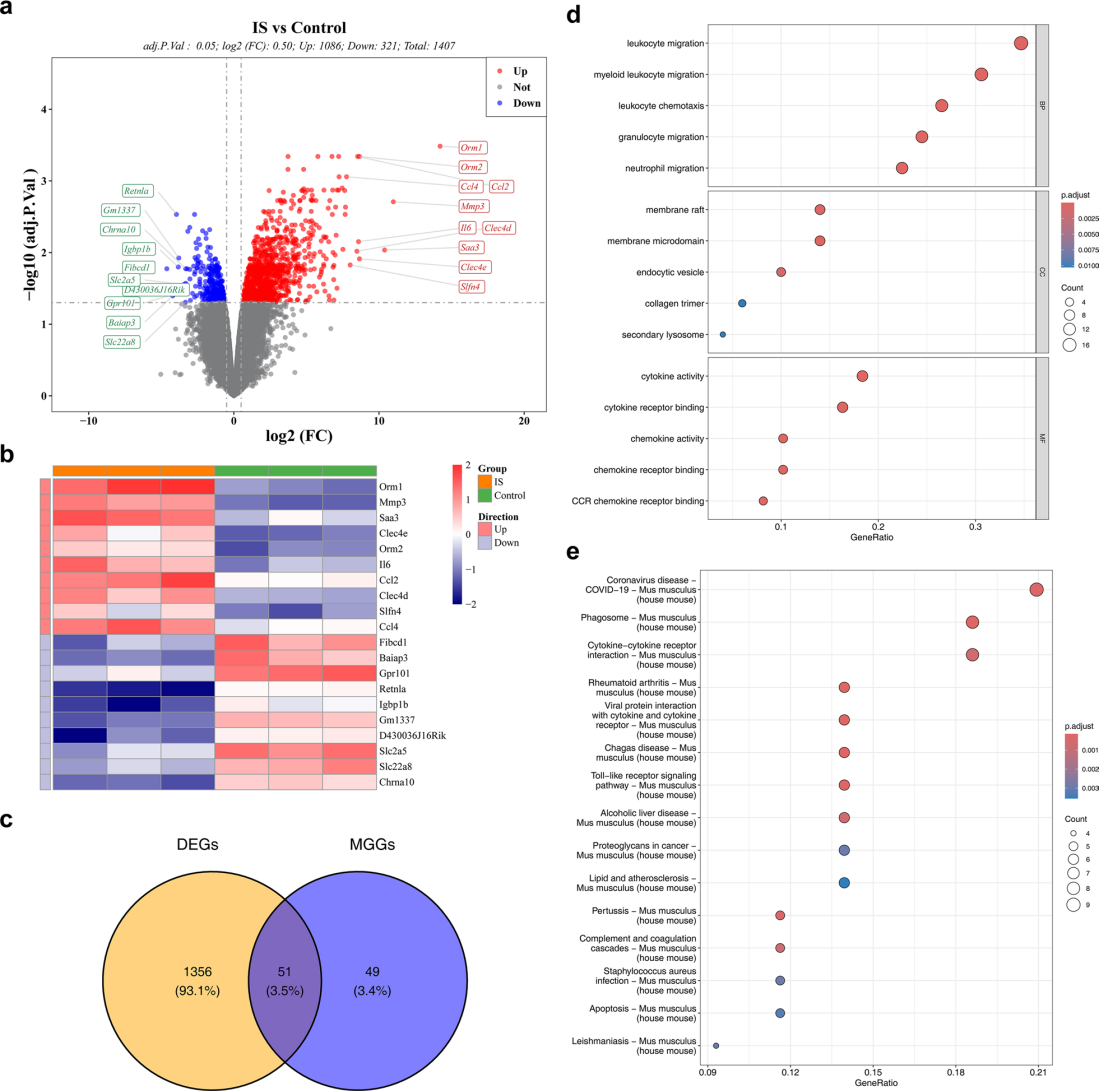
Screening and identification of candidate genes. **a** Volcanic plot of differentially expressed genes distribution between IS and Control, abscissa Log2FC, ordinate − Log10 (adjP. Value), each dot represents a gene; The transverse reference line represents −  Log10(0.05) = 1.3, the longitudinal reference line represents log2FC =  ± 0.5, and the genes in the upper right corner are up-regulated differentially expressed genes (indicated by red), the genes in the upper left corner are down-regulated differentially expressed genes (represented by blue), and the rest of the genes are genes with no significant statistical significance (represented by gray). The genes labeled in the figure were the top 10 up-regulated genes and the top 10 down-regulated genes with the largest log2(FoldChange)||, i.e., the largest fold of difference. **b** Differential gene expression heat map, annotation bar above, orange for disease samples, green for control samples; The ordinates in the heat map represent genes, with red being the most expressed genes and blue being the low expression genes. **c** Venn diagram of candidate genes. **d** GO enrichment analysis results. **e** KEGG enrichment analysis results

Differential expression analysis revealed that there were 1407 DEGs between the IS group and the control group. Among them, in the IS group, 1086 genes were up-regulated genes, and 321 genes were identified as down-regulated genes (Fig. 4a). The volcano plot marked the top 10 genes with the most significant up-regulation and down-regulation (ranked from high to low according to |log_2_FC|). In addition, a heatmap was used to illustrate the expression profiles of the above-mentioned genes (Fig. 4b). Subsequently, an intersection analysis was performed on the 1407 DEGs and the 100 MGGs, and finally 51 candidate genes were identified (Fig. 4c). Subsequently, an enrichment analysis was carried out to understand the signaling pathways involved by the candidate genes. The candidate genes were significantly enriched in 571 GO terms (adj.*p* < 0.05), comprising 517 biological processes (BPs), 13 cellular components (CCs), and 41 molecular functions (MFs) (Fig. 4d) (Additional file 1). In particular, the top 3 BP terms included leukocyte migration, myeloid leukocyte migration, and leukocyte chemotaxis. Among the CCs, terms such as membrane raft, membrane microdomain, and endocytic vesicle played prominent roles. Meanwhile, regarding molecular functions (MFs), they were mainly enriched in cytokine activity, cytokine receptor binding, and chemokine activity. The GO analysis findings showed that the candidate genes were crucial for functions such as clearing necrotic tissue, initiating repair, and promoting nerve regeneration in IS disease. In addition, the KEGG enrichment analysis of the candidate genes demonstrated that among the top 15 significantly enriched pathways, pathways such as coronavirus disease-mus musculus (COVID-19), phagosome-mus musculus, cytokine-cytokine receptor interaction-mus musculus, and rheumatoid arthritis-mus musculus were significantly associated with the candidate genes (Fig. 4e) Additional file 2). This suggested that the candidate genes exerted crucial functions in the onset, progression, and prognosis of IS.

We intersected the human homologs of 51 candidate genes from mice with risk genes for age-related neurological diseases, resulting in the identification of 9 intersecting genes, namely CD14, CTSB, EMP3, LGALS1, LGALS3, MSR1, RCAN1, SPP1 and TUBA1C. This finding suggested that these six genes might be involved in age-related functions or disease-related abnormal activation of microglia in the human brain (Additional file 3).

## 5Cd14, Csf1 and Tlr2 were identified as key genes for IS

**Fig. 5:**
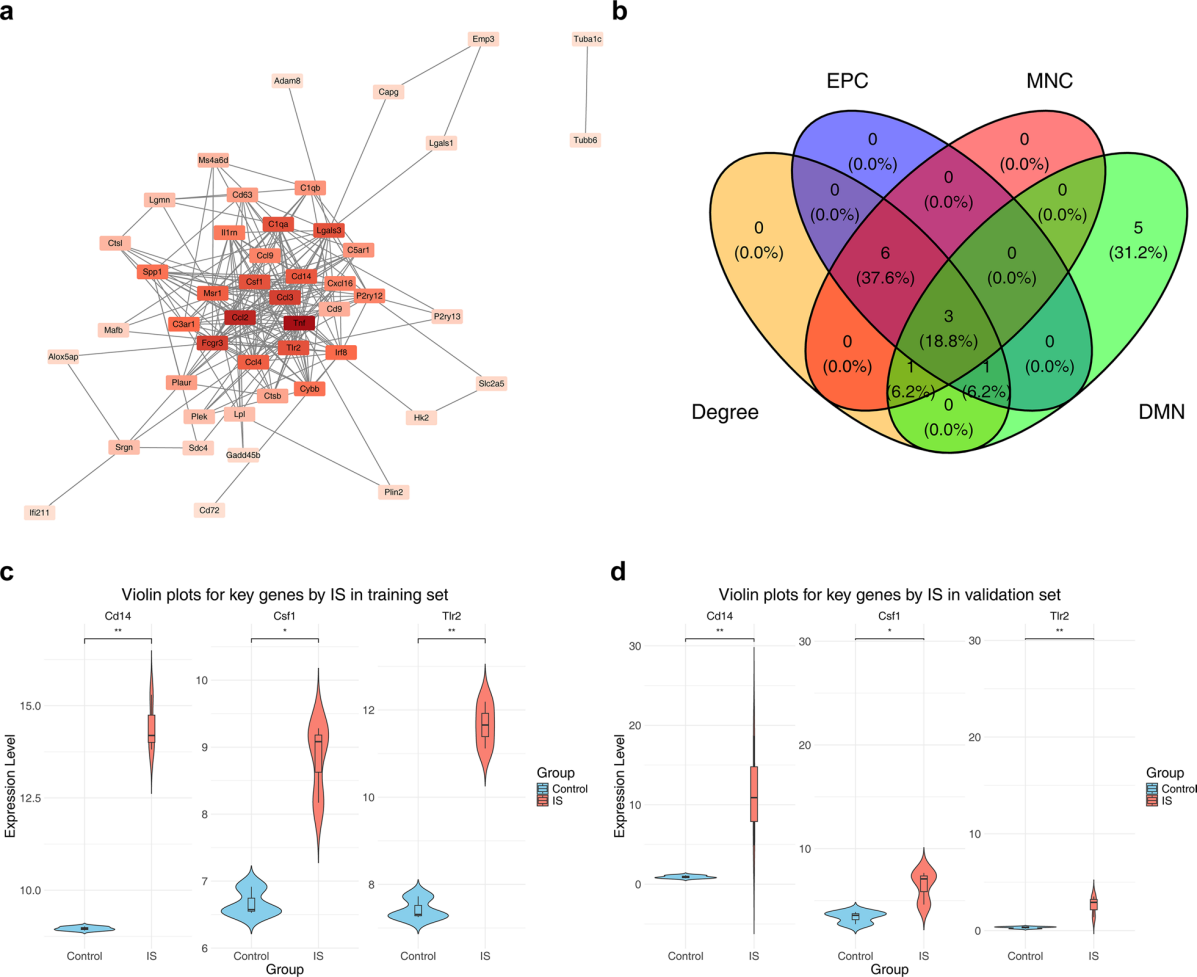
PPI network construction and expression level verification of candidate genes. **a** PPI network of candidate genes, the shade of color represents the degree value of the gene, the darker the color, the higher the degree value of the gene, the more genes that interact with the gene, the lighter the color, the smaller the degree value of the gene, and the fewer genes that interact with the gene. **b** cytoHubba screening Venn diagram, **c** Expression levels of candidate key genes in the IS group and control group in the training set. **d** Expression levels of candidate key genes in the IS and control groups in the validation set

Subsequently, a PPI network consisting of 255 interaction relationships corresponding to 47 candidate genes was constructed (Fig. 5a), and 4 genes formed isolated targets. Among the network, Tnf, Ccl2, Fcgr3, Ccl3, and Lgals3 had frequent protein-level interactions with other genes. After that, these candidate genes were integrated into four algorithms of the cytoHubba plugin. Cd14, Csf1, and Tlr2 were obtained from the intersection of the top 10 genes in each algorithm (Fig. 5b). Then, in the IS groups and control groups of GSE58720 and GSE202659, the expression levels of Cd14, Csf1, and Tlr2 increased significantly (*p* < 0.05), and a consistent expression trend was witnessed in the two datasets (Fig. 5c, d). This indicated their potential value in the diagnosis of IS, and they were regarded as key genes.

### GSEA and GSVA of Cd14, Csf1 and Tlr2

**Fig. 6:**
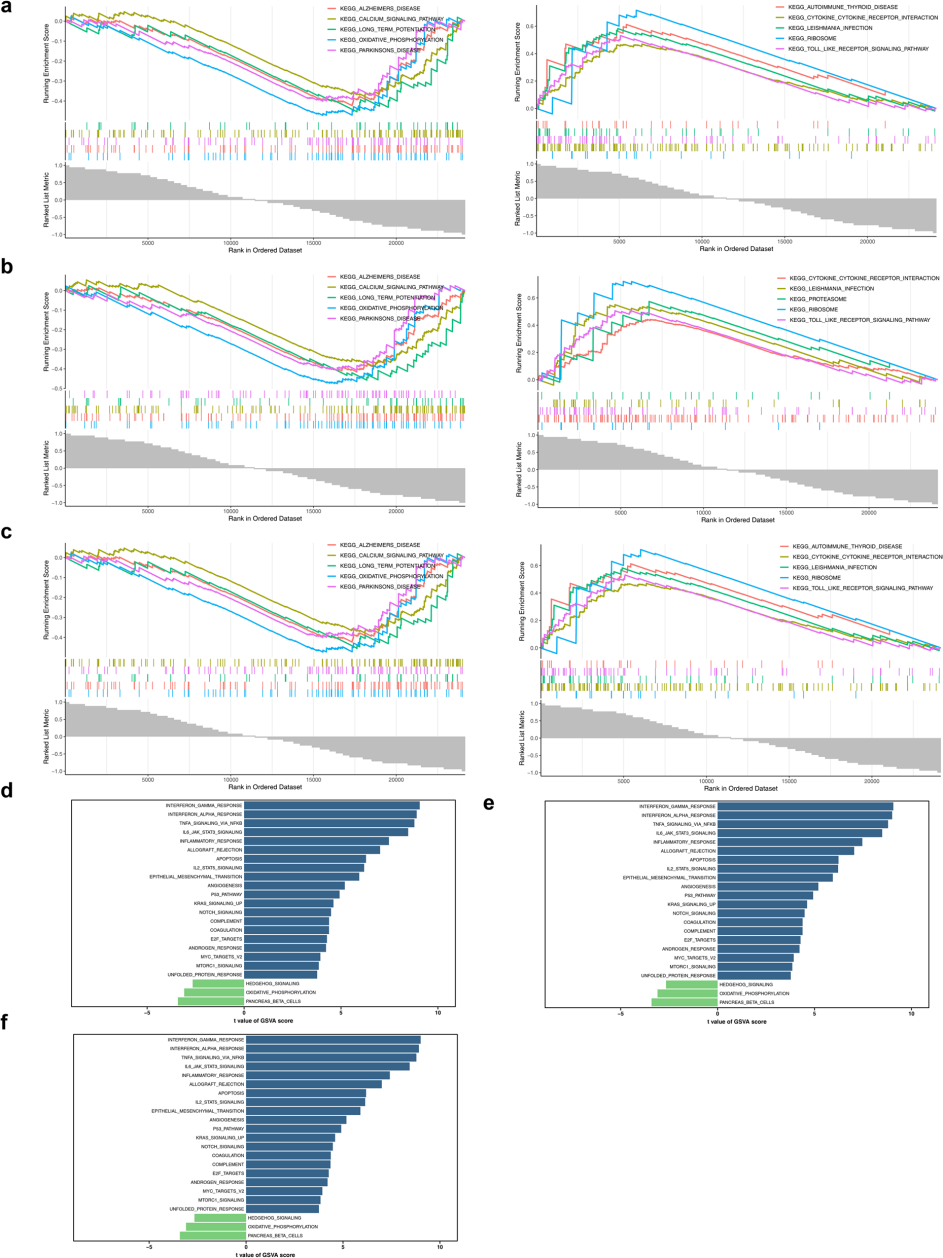
Enrichment analysis of key genes. **a** GSEA analysis of CD14. **b** GSEA analysis results for Csf1. **c** GSEA analysis of Tlr2, the upper part represents the enrichment fraction of the TOP5 gene pathway, the middle part represents the distribution of genes in the reference gene set in the training set, different colors represent different pathways, and the lower part represents the signal-to-noise ratio, where the larger the area, the greater the multiple of the difference. **d** GSVA analysis results for CD14. **e** GSVA analysis results for Csf1. **f** GSVA analysis results of TLR2, the ordinate represents the pathway name of the differential pathway, the abscissa represents the differential pathway GSVA score, the green bar chart represents the down-regulated pathway, and the blue bar chart represents the up-regulated pathway

Among the top 5 up-regulated and down-regulated pathways that were significantly enriched in Cd14, Csf1, and Tlr2, the pathways that were commonly enriched by the 3 genes included cytokine-cytokine receptor interaction, leishmania infection, ribosome, and toll-like receptor signaling pathway (Fig. 6a–c). Tlr2 and Cd14 were co-enriched in the calcium signaling pathway, long-term potentiation, oxidative phosphorylation, and toll-like receptor signaling pathway. The co-enrichment suggested that Cd14, Csf1, and Tlr2 might participate in disease regulation in areas such as inflammation and immune regulation, energy and substance metabolism, and neural function regulation. GSVA analysis showed that among the pathways significantly and differentially enriched between the disease group and the control group were interferon gamma response, interferon alpha response, TNF-α signaling via NF-κB, IL-6 JAK-STAT3 signaling, inflammatory response, allograft rejection, hedgehog signaling, TGF-β signaling, and G2/M checkpoint, etc. These pathways play roles in the development of IS mainly through aspects including immune and inflammatory regulation, regulation of cell biological processes, and metabolic regulation (Fig. 6d–f).

### Cd14, Csf1 and Tlr2 were regulated by multiple factors

**Fig. 7:**
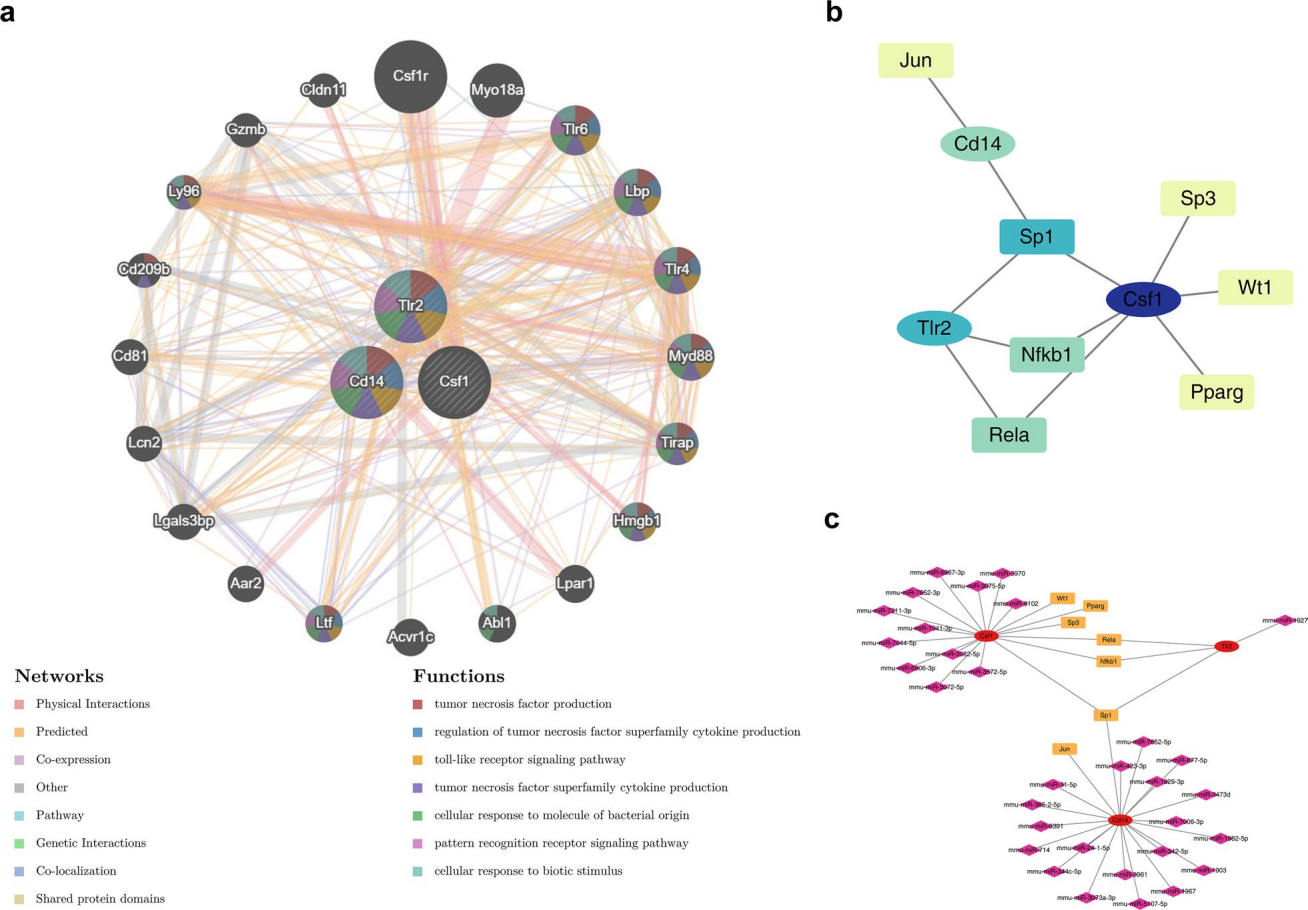
Construction of regulatory networks of key gene-related molecules. **a** Interaction network between key genes and co-expressed genes. **b** Regulatory network of key genes and transcription factors. **c** Regulatory network between biomarkers and TF and miRNAs, with lines indicating regulatory relationships between key genes and TF and miRNAs. Different colors represent different factors, with yellow rectangles for TFs, pink diamonds for miRNAs, and ovals for key genes

Further research on the regulatory factors of Cd14, Csf1, and Tlr2 revealed a total of 7 TFs that regulate the key genes (Fig. 7a). Among them, Sp1 was observed to target all 3 key genes. Additionally, Csf1 was regulated by 1,054 miRNAs, Cd14 by 35 miRNAs, and Tlr2 by 1 miRNA, such as mmu-miR-3072-5p, mmu-miR-3970, mmu-miR-6906-3p, mmu-miR-7041-3p, mmu-miR-7652-3p, mmu-miR-3572-5p, mmu-miR-7044-5p, and mmu-miR-1927. A regulatory network was constructed based on the TFs, key genes, and the 20 miRNAs with the lowest predicted binding *p *values. The network showed that a total of 32 miRNAs and TFs jointly regulate the key genes, with 43 types of interactions among them (Fig. 7b). This indicates that Cd14, Csf1, and Tlr2 were regulated by multiple factors. Meanwhile, genes with similar functions to the key genes were predicted. Different interaction patterns were formed among the 20 genes, and these interactions were associated with functions such as tumor necrosis factor production, tumor necrosis factor superfamily cytokine production, cellular response to molecule of bacterial origin, and pattern recognition receptor signaling pathway (Fig. 7c).

### Drug prediction and molecular docking of Cd14, Csf1 and Tlr2

**Fig. 8:**
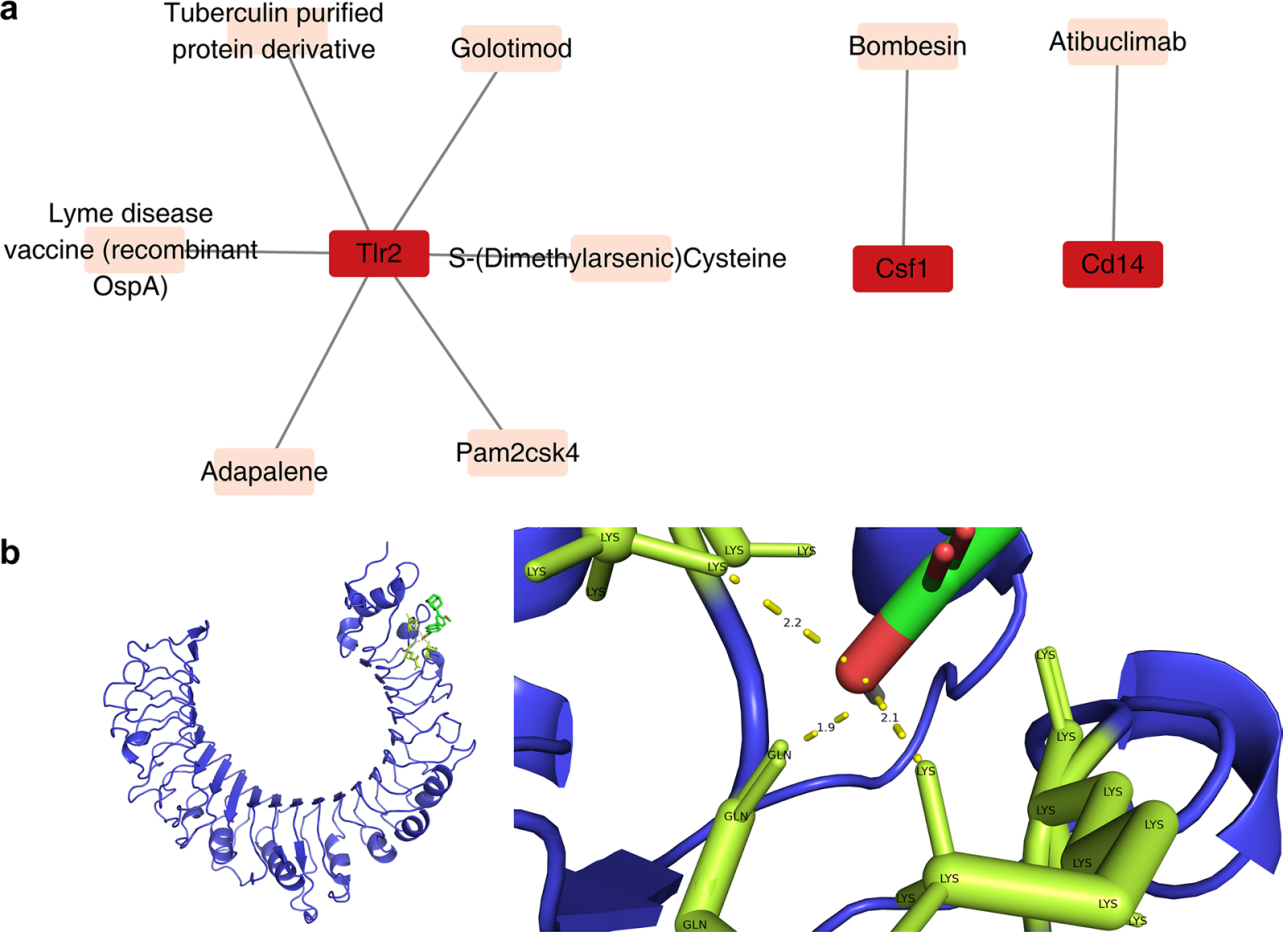
Drug prediction of key genes. **a** Network diagram of key genes and potential therapeutic drugs. **b1-2** Molecular docking of the predicted drug to a potential target protein of a key gene

The drug-prediction results showed that 6 drugs were predicted for Tlr2, such as Tuberculin purified protein derivative; 1 drug, Atibuclimab, was predicted for Csf1; and 1 drug, Bombesin, was predicted for Cd14 (Fig. 8a). The molecular formulas and structures of the 8 drugs were shown in Table 1. Among the 8 drugs, 6 drugs could not be downloaded in SDF format or failed in docking. Finally, only 2 drugs were subjected to molecular docking. The free-binding energy between Golotimod and Tlr2 was − 3.96 kcal/mol, and the free-binding energy between Adapalene and Tlr2 was -9.73 kcal/mol. Therefore, Adapalene showed a very good binding affinity with Tlr2. The results of the molecular docking were shown in Fig. 8b. Amino acid residues such as lysine (LYS) and glutamine (GLN) were likely to be involved in the interaction with the ligand.

**Table 1:** Structure of the drug molecule

Pubchem ID	Molecular formula	2D structure
17753880	C_5_H_12_AsNO_2_S	
9989023	C_65_H_126_N_10_O_12_S	
6992140	C_16_H_19_N_3_O_5_	
60164	C_28_H_28_O_3_	
16201612	C_71_H_110_N_24_O_18_S	

### Validation of Cd14, Csf1 and Tlr2

**Fig. 9:**
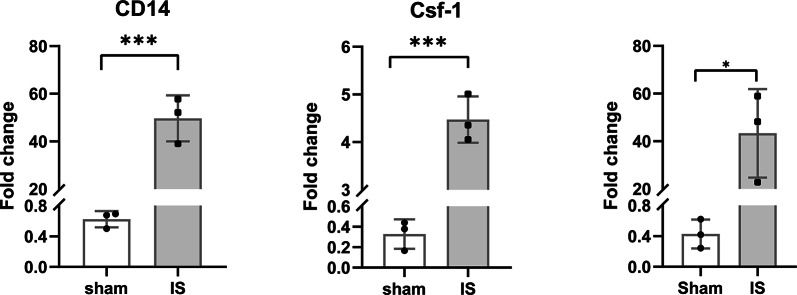
qRT-PCR detection of key genes, ***p < 0.001

The gene expression difference was veriffed by qRT-PCR measurement of 6 samples of tMCAO and sham mice from Shang Hai Jiao Tong University and Fudan university. The results showed that Cd14, Csf1 and Tlr2 were highly expressed in tMCAO brain tissues. The results were consistent with the TCGA database (Fig. 9).

## Discussion

The pathogenesis of IS is intricately linked to microglial activation, which modulates neuroinflammation and neuronal damage through immune signaling pathways [2]. This study integrated transcriptomic datasets of tMCAO mice from the GEO database to explore the regulatory roles of MGGs. Through integrated analysis of single-cell and bulk RNA sequencing data, 51 candidate genes were identified. Subsequent functional enrichment analysis and PPI network construction pinpointed three key genes: Cd14, Csf1, and Tlr2. Furthermore, GeneMANIA analysis and GSEA elucidated the regulatory relationships of these key genes and identified potential drug targets, providing novel insights for the diagnosis and treatment of IS.

The Cluster of Differentiation 14 (Cd14) gene is located on chromosome 18 in mice. Similar to its human counterpart, the murine Cd14 gene comprises multiple exons and introns [10]. This structural feature allows the gene to undergo alternative splicing during transcription, generating multiple mRNA isoforms that may translate into protein isoforms with distinct functional properties. As a pattern recognition receptor, Cd14 specifically recognizes pathogen-associated molecular patterns and plays a pivotal regulatory role in inflammatory responses. Upon recognition of pathogens by cell-surface Cd14, immune cells release inflammatory cytokines that initiate anti-pathogen inflammatory cascades [11, 12]. Notably, Cd14 has also been implicated in IS pathogenesis. Cd14 upregulation on activated microglia drives neuroinflammation through MyD88/NF-κB-mediated pathways, inducing iNOS/NO overproduction and TNF-α/IL-1β release, which reciprocally enhance Cd14 activity and neuronal apoptosis in IS [13]. Beyond local injury, Cd14 + extracellular vesicles from activated monocytes/microglia propagate systemic inflammation, correlating with transient ischemic attack (TIA) diagnosis and cardiovascular risk [14, 15]. Therapeutic targeting via Cd14 silencing attenuates neurotoxicity in preclinical models but risks impairing TLR4-TRIF-dependent bacterial clearance [12]. Precision strategies, such as temporal modulation or compartment-specific targeting (EV neutralization), combined with Tlr2/4 inhibitors, may balance anti-inflammatory efficacy and microbial defense.

The Colony Stimulating Factor 1 (Csf1) gene is located on mouse chromosome 3. Through alternative splicing, it generates multiple mRNA isoforms encoding distinct Csf1 glycoprotein variants. These proteins exert their biological effects by specifically binding to the Csf-1 receptor (Csf-1R) on the cell surface [16]. In IS, Csf1 exhibits dual-phase roles: acutely, it recruits monocytes and activates microglia, driving neuroinflammation and oxidative injury [17, 18]; however, delayed Csf1 administration enhances neuroprotection by suppressing pro-inflammatory cytokines and restoring CREB-dependent synaptic plasticity [19, 20]. Csf1 also promotes neural repair through neural stem cell proliferation and angiogenesis, though elevated Csf1 levels correlate with stroke risk, potentially via pathological vascular interactions [18, 21]. For instance, Csf1 overexpression exacerbates BBB disruption via sLOX-1 upregulation, reversible by dual inhibition [22]. Therapeutic Csf1R blockade (e.g., PLX5622) mitigates chronic neuroinflammation but risks impairing homeostatic microglial functions [17]. Precision strategies, such as temporally restricted inhibition (acute phase) or cell-specific targeting (e.g., neuronal Csf1), may dissociate detrimental synaptic pruning from beneficial tissue repair, balancing neuroprotection with physiological microglial activity [23].

The Toll-Like Receptor 2 (Tlr2) gene resides on mouse chromosome 4. Through alternative splicing during transcription, this gene generates multiple mRNA isoforms that may encode protein variants with distinct functional properties. Tlr2 encodes a transmembrane protein-Tlr2 capable of either specifically recognizing pathogen-associated molecular patterns or interacting with downstream signaling molecules to activate immune pathways. Tlr2 is a transmembrane receptor mediating neuroinflammation in IS via MyD88-dependent DAMP recognition [24]. Post-ischemia, Tlr2 activation on microglia and neurons triggers divergent pathways: (1) neuronal MyD88/JNK-AP1 signaling induces caspase-3-dependent apoptosis through calcium dysregulation [25]; (2) microglial Tlr2/Sphk1 axis amplifies IL-1β/TNF-α release via S1P-mediated feedback, exacerbating neuroinflammation [26]. Paradoxically, Tlr2 deficiency shifts ischemic cell death from apoptosis to necrosis, underscoring its dual role in death modality regulation [25]. Clinically, elevated blood Tlr2 mRNA correlates with severe neuroinflammation and poor prognosis [27]. Pharmacological Tlr2/4 inhibition (e.g., JLX001) attenuates NF-κB activation, microglial hyperreactivity, and oxidative stress, preserving neuronal survival [28]. However, complete Tlr2 blockade risks compromising antimicrobial defenses, necessitating spatiotemporal therapeutic strategies—early-phase inhibition (0–72 h) to suppress neuroinflammation versus later-phase restoration to support repair processes.

Our findings revealed that the Cd14/Csf1/Tlr2 genes collectively demonstrated significant enrichment in six key pathways: cytokine-cytokine receptor interaction, Leishmania infection, ribosome biogenesis, Toll-like receptor signaling pathway, interferon gamma response, and Hedgehog signaling pathway. Cytokine-cytokine receptor interaction pathway serves as a nexus for amplifying ischemic damage through pro-inflammatory cascades. Tlr2 and Cd14 jointly potentiate cytokine release (e.g., TNF-α, IL-1β) via MyD88/NF-κB signaling, while Csf1 modulates macrophage polarization to regulate cytokine balance [29, 30]. Notably, m6A hypomethylation in IS patients enhances cytokine receptor transcription, exacerbating neuroinflammation [29]. Targeting this axis may require dual inhibition of upstream DAMPs recognition (via Tlr2/Cd14) and downstream Csf1-driven macrophage recruitment. Ischemia induces dynamic ribosome reprogramming to prioritize stress-responsive protein synthesis. Upregulated ribosomal proteins (e.g., RPS23) facilitate translation of repair factors like HSP70 in astrocytes, while OGFOD1-mediated hydroxylation links ribosomal stress to the UPR activation [31, 32]. Csf1 may indirectly regulate ribosomal activity by enhancing microglial phagocytosis of damaged neurons, thereby reducing proteotoxic burden. Therapeutic strategies targeting ribosome quality control (e.g., FG4592) could synergize with CSF1R inhibitors to restore proteostasis.

Tlr2 and Cd14 form a co-receptor complex that amplifies DAMP recognition, driving MyD88-dependent neurotoxicity and Sphk1-mediated cytokine storms [33, 34]. Csf1 intersects this pathway by sustaining microglial survival, which perpetuates Tlr2/Cd14 signaling. Pharmacological disruption of Tlr4/NF-κB (e.g., NXT capsules) shows promise in attenuating gut-brain axis inflammation, suggesting combinatory approaches targeting Tlr2-Cd14-Csf1 crosstalk may enhance efficacy [35]. IFN-γ exacerbates ischemic injury by skewing microglia toward M1 polarization, a process potentiated by Tlr2-mediated STAT1 activation and Csf1-dependent macrophage accumulation [36]. Paradoxically, IFN-γ also primes antigen presentation for tissue repair. Spatiotemporal modulation—suppressing early IFN-γ while promoting its delayed immunoregulatory effects—could exploit this duality. The Sonic Hedgehog (Shh) pathway counterbalances neuroinflammation by promoting M2 microglial polarization and angiogenesis [37]. Csf1 synergizes with Shh via PDGFA-mediated fibroblast activation, facilitating fibrotic scar formation. Tlr2 inhibition may further enhance Shh-driven repair by reducing pro-inflammatory TGF-β1 suppression [38]. Small-molecule Shh agonists (e.g., NBP) represent viable adjuvants to CSF1R-targeted therapies. While no direct evidence links this pathway to IS, its enrichment suggests shared immune evasion mechanisms.

Our analysis delineates a regulatory triad involving miR-3072-5p, miR-3970, miR-1927, NF-κB (Nfkb1/Rela), which orchestrates vascular repair, neuroinflammation, and cellular stress responses in IS.miR-3072-5p acts as a brake on angiogenesis by targeting VEGF’s 3′UTR, suppressing its translation under physiological conditions [39]. Remote ischemic preconditioning (rIPC) alleviates spinal ischemia through miR-3072-5p downregulation, which derepresses VEGF to enhance perfusion and neuronal survival. This mechanism may extend to IS, where miR-3072-5p silencing could amplify the collateral circulation in penumbral regions. However, excessive VEGF elevation risks BBB disruption, necessitating spatiotemporal control via nanoparticle-delivered antagomirs.Ischemia-triggered IKK activation phosphorylates IκBα, liberating Rela (p65) to translocate into the nucleus and initiate transcription of TNF-α, IL-1β, and iNOS [40, 41]. Pharmacological inhibitors (e.g., DZSM) and siRNA strategies (e.g., p65/CMI) effectively block Rela’s nuclear import, attenuating M1 microglial polarization and neuronal apoptosis [41, 42]. Paradoxically, TLR2 activation by Pam2CSK4 engages MyD88-dependent NF-κB signaling to induce a “hybrid” microglial phenotype—simultaneously enhancing debris clearance (via IL-1β) and tissue repair (via IL-10) [43]. This duality suggests NF-κB’s role is phase-dependent: early inhibition mitigates acute inflammation, while timed activation in subacute phases may promote resolution. Despite their co-enrichment in IS-related pathways, no direct evidence links these miRNAs to IS pathophysiology. Given that miR-3970’s has predicted binding sites in NF-κB inhibitors (e.g., IκBα), and miR-1927’s potential regulation of TLR2, further studies should explore their roles in fine-tuning neuroinflammatory cascades.

Our computational drug prioritization identified Adapalene as the top candidate targeting Tlr2, demonstrating superior binding affinity and multimodal neuroprotective potential. As a third-generation retinoid, Adapalene suppresses Tlr2/NF-κB-driven neuroinflammation by inhibiting lipoxygenase-mediated arachidonic acid metabolism while concurrently activating RAR-β to enhance neuronal antioxidant defenses, as evidenced in ALS models where its nanoformulation (Adap-NPs) crosses the blood–brain barrier; prolongs survival, and mitigates oxidative apoptosis [44, 45]. This dual action—attenuating MyD88-dependent cytokine storms and stabilizing vascular integrity—aligns with ischemic stroke pathophysiology, particularly in subacute phases where controlled Tlr2 inhibition may balance inflammatory resolution and angiogenesis. While other candidates (e.g., Pam2CSK4) exhibit Tlr2 agonism, their thrombotic risks and lack of CNS specificity limit translational feasibility [46]. Future studies should optimize Adapalene’s spatiotemporal delivery using microglia-targeted nanocarriers and validate its efficacy in preclinical stroke models, particularly in combination with VEGF-enhancing strategies to synergize vascular repair and neuroprotection. Other compounds—S-(Dimethylarsenic) cysteine, Pam2CSK4, tuberculin purified protein derivative, recombinant OspA Lyme disease vaccine, Golotimod, Atibuclimab, and Bombesin—have been minimally studied in the context of neurological and vascular diseases, warranting further investigation.

To corroborate our bioinformatic predictions, we performed qRT-PCR analysis on ipsilateral cortical tissues from C57BL/6 mice subjected to tMCAO with 3-day reperfusion. qRT-PCR results demonstrated significantly higher expression of Csf1, Tlr2, and Cd14 in ischemic brains compared to sham-operated controls, consistent with the bioinformatic analysis.

This study highlights MGGs, yet the pathogenesis of IS depends not on microglia alone but on the coordinated “vascular–microglia axis,” involving endothelial cells, smooth muscle cells, and pericytes [47]. Dysfunction of this axis contributes critically to IS. Endothelial injury [48], aberrant smooth muscle proliferation [47], and pericyte dysfunction [49] underlie vascular pathology. For instance, endothelial ICAM-1 upregulation promotes leukocyte infiltration [50], while VEGF secretion facilitates vascular repair [47]. Smooth muscle MMP9 activation further compromises vascular integrity [51]. KEGG analysis of 51 candidate MGGs revealed enrichment in the “cytokine–cytokine receptor interaction” pathway, consistent with these vascular processes. Both ICAM-1 and MMP9 are regulated through this pathway [52, 53]. Notably, Csf1 has been identified as a key mediator of endothelial–macrophage crosstalk and angiogenesis [54]. Given that Csf1 emerged as a hub MGG, we propose that microglia-derived Csf1 may act on endothelial CSF1R to induce VEGF release and vascular repair, while also influencing endothelial ICAM-1 and smooth muscle MMP9 via cytokine–receptor signaling. Although this mechanism remains unverified, future studies employing iPSC-derived endothelial or smooth muscle cells co-cultured with microglia could clarify the vascular–microglia axis, offering experimental support for novel therapeutic strategies targeting IS.

Recent large-scale whole-genome sequencing (WGS) studies of Alzheimer’s disease (AD) have identified microglia and immune-related pathways as central regulators of disease risk. Genes such as APOE, APCDD1, and SAMD3 modulate AD progression by influencing microglial phagocytosis of amyloid-β and inflammatory cytokine release [55]. These findings are consistent with our GSEA/GSVA results, which implicate Cd14, Csf1, and Tlr2 in regulating neuroinflammation and immune responses via the “cytokine–cytokine receptor interaction” and “Toll-like receptor signaling” pathways. Although the specific genes differ, both IS and AD converge on the common mechanism of microglia-mediated immune regulation, suggesting a conserved strategy of microglial immune adaptation in response to diverse CNS insults such as ischemia or protein deposition. Notably, Tlr2 has been shown in Parkinson’s disease to promote prion-like propagation of protein aggregates through microglial activation [56]. Similarly, cerebral ischemia rapidly induces protein aggregation, exacerbating IS pathology, raising the possibility that Tlr2 may act in IS through analogous mechanisms [57]. Distinctly, however, our findings suggest that Cd14, Csf1, and Tlr2 extend beyond immune signaling to participate in vascular remodeling [54, 58, 59]. This “vascular–microglia axis” differs fundamentally from the proteinopathy-driven microglial activation observed in AD [60], pointing to a vascular-linked dimension of immune regulation unique to IS. Nevertheless, this study did not directly compare the expression and function of Cd14, Csf1, and Tlr2 between IS and other neurodegenerative disorders, leaving their potential connection to proteinopathy unresolved. Future work should address these differences, delineate IS-specific microglial pathways, and investigate the roles of Cd14, Csf1, and Tlr2 across CNS diseases, thereby providing new insights for translational research spanning vascular and protein aggregation pathologies.

Recent reviews emphasize that genetic and epigenetic instability-including DNA methylation drift, histone modification changes, and clonal hematopoiesis (e.g., DNMT3A, TET2 mutations)-is a hallmark of aging that increases cellular vulnerability to injury [61]. Given the higher incidence of IS in the elderly [62], overlap between our candidate genes and age-associated epigenetic programs in microglia could contextualize our findings within the broader framework of aging. By intersecting 51 microglia-related candidates with human age-related neurological risk genes, we identified nine core genes (CD14, CTSB, EMP3, LGALS1, LGALS3, MSR1, RCAN1, SPP1, TUBA1C), suggesting a potential link between aging, microglial function, and CNS disease. Mechanistically, aging is associated with global DNA hypomethylation and dysregulated expression of epigenetic modifiers, which may contribute to microglial dysfunction [63]. Aged microglia also show upregulation of inflammation-related genes [64]. Notably, key regulators identified in this study (Tlr2, Csf1) are central inflammatory mediators [65, 66]. This implies that age-related epigenetic dysregulation may amplify microglial pro-inflammatory responses by enhancing expression of such genes, thereby exacerbating neuroinflammation in IS. Importantly, Tlr2 and Csf1 may form an interaction network with aging-associated epigenetic programs, driving inflammatory signaling and creating a vicious cycle of “aging–epigenetic dysregulation–inflammation amplification,” which could accelerate IS progression. Although these interpretations remain speculative, future work using single-cell epigenomic approaches in young and aged IS models will be crucial to validate the roles of these genes and their epigenetic regulation. Such studies may provide a basis for developing age-specific therapeutic strategies for elderly IS patients.

This study employed bioinformatics approaches to comprehensively analyze IS and successfully identified three microglia-related key genes: Cd14, Csf1, and Tlr2, which were further validated in an animal model via qRT-PCR. While the current research provides novel bioinformatics perspectives and evidence for pivotal genes in IS, enhancing the credibility of these candidates necessitates subsequent experimental investigations in animal and cellular models to elucidate their specific biological roles and functional mechanisms in IS pathophysiology.

## Materials and methods

### Data origin

In this study, the datasets were retrieved from the Gene Expression Omnibus (GEO) database (https://www.ncbi.nlm.nih.gov/geo/). The dataset GSE58720 (platform: GPL10787) was utilized for the training set, consisting of 3 brain tissue samples from middle cerebral artery occlusion (MCAO) mice and 3 samples from sham-operated mice (control group). The GSE202659 dataset (platform: GPL24247) served as the validation set, containing 3 MCAO mice brain tissue samples and 3 sham-operated control samples. Additionally, the single-cell dataset GSE174574 (platform: GPL21103) included 3 MCAO mice brain tissue samples and 3 sham-operated control samples.

### Processing of the scRNA-seq data

In the GSE174574 dataset, the Seurat package (v 5.1.0) was utilized for quality control (quality control criteria: 200 < nFeature_RNA < 6000, percent.mt < 15%, nCount_RNA < 20,000, and filtered out cells with fewer than 200 genes and genes covered by fewer than 3 cells) [67]. Furthermore, the FindVariableFeatures function in the Seurat package (v 5.1.0) was used, and according to the variance stabilization transformation (vst) method, the top 2000 genes with relatively high coefficients of variation between cells, namely highly variable genes (HVGs) were obtained, and the top 10 HVGs with the most variation were identified by LabelPoints function and labeled in this study. Similarly, principal components analysis of HVGs was performed using the Seurat package (v 5.1.0). RunPCA function, and the Elbowplot and JackStraw function were employed to plot the scree plot to show the contribution of the top-ranked principal components (PCs) to cellular variation and quantify the significance of the PCs, respectively (*p* < 0.05). After completing the PCA dimensionality reduction, the Seurat package (v 5.1.0) (resolution = 0.2) was utilized to cluster the cells utilizing the uniform manifold approximation and projection (UMAP) method. Subsequently, the FindAllMarkers function of the Seurat package (min.pct = 0.25, logfc.threshold = 0.25, test.use = auc) was employed to detect the differentially expressed genes in each cell cluster. The Marker genes reported in previous studies were used as the primary reference, and the CellMarker dataset (http://117.50.127.228/CellMarker/) was used as the secondary reference to annotate the cell populations [68]. A bubble plot was drawn to visually display the representation of Marker genes in various cell types. After annotation, a UMAP plot of different cell type groups was drawn for visualization.

### Pseudotime analysis and cellular communication analysis

Aiming to understand the differentiation trajectory of microglia during the disease-onset process, the RunPCA, FindNeighbors, and FindClusters functions in the Seurat package (v 5.1.0) were sequentially used to perform dimensionality reduction and clustering on microglia, which were then annotated into different subgroups (*p* < 0.05, resolution = 0.2). Subsequently, the ggplot2 package (v 3.5.1) was employed to explore the proportional distribution of cells in each subgroup within disease samples and control samples [69]. Next, the Monocle2 package (v 2.28.0) was utilized to analyze the differentiation trajectory [70]. In addition, to analyze the communication between microglia and other cells in IS, based on the CellChatDB database (http://www.cellchat.org/cellchatdb/), with the “CellChatDB.mouse” as a reference, the aggregateNet function of the CellChat package (v 1.6.1) was utilized to analyze the cell-to-cell communication of ligand-receptor complexes (mean = trimean, trim = 0.1) [71]. Moreover, based on functions such as identifyOverExpressedGenes, identifyOverExpressedInteraction, ProjectData, computeCommunProb, filterCommunication, and computeCommunProbPathway, potential ligand-receptor interactions were identified, and an interaction analysis was conducted on the cell subgroups within microglia (*p* ≤ 0.05, log_2_ mean (Molecule 1, Molecule 2) ≥ 0.1).

### Identification of MGGs

Furthermore, to obtain genes associated with microglia in IS, for the GSE174574 dataset, the MetacellsByGroups function in the hdWGCNA package (v 0.4.00) was applied to construct metacells (50 cells) based on the microglia subgroups of each sample [67]. The TestSoftPowers function was employed to select an appropriate soft threshold, and based on this soft threshold, the ConstructNetwork function was utilized to create a weighted co-expression network. Subsequently, the hierarchicalCluster function was applied for hierarchical clustering (gene modules ≥ 50) to obtain co-expressed gene modules. The ModuleEigengenes function was used to calculate the gene correlations within each module, and the ModuleConnectivity function was applied to evaluate the connectivity between modules. Then, the gene expression levels of each module were compared between disease and control samples using Wilcoxon test (*p* < 0.05). Modules with significantly altered gene expression abundances were selected, and the genes within these modules were regarded as MGGs.

### Uncovering and analyzing the functions of candidate genes

To obtain genes that exhibited differential expression in the IS and control groups within the GSE58720 dataset, the limma package (v 3.58.1) was employed to discern DEGs(|log_2_Fold change (FC)|> 0.5, adj.*p* < 0.05) [71]. Moreover, the volcano plot and heatmap of DEGs were produced via the ggplot2 package (v 3.5.1) and the ComplexHeatmap package (v 1.0.12), respectively [69, 72]. The intersection of DEGs and MGGs was determined by means of the ggvenn package (v 0.1.10) (https://CRAN.R-project.org/package=ggvenn) to identify candidate genes associated with IS and microglia. Subsequently, the candidate genes were analyzed for Gene ontology (GO) and Kyoto Encyclopedia of Genes and Genomes (KEGG) analyses (adj.*p* < 0.05) via the clusterProfiler package (v 4.8.3), thus elucidating the biological functions of the candidate genes [73].

To clarify the association between microglia-related candidate genes and age-related neurological diseases in the human brain, this study utilized publicly available single-cell atlas data of human brain tissue after death for analysis. The specific process was as follows: From the Zenodo database [74], which contains 6761 gene expression profile datasets related to the risk of age-related neurological diseases, the mouse candidate genes in this study were first converted into their corresponding human homologous genes. Then, an intersection screening was conducted between the human homologous genes and the age-related neurological disease risk genes in the dataset. Finally, the R package “ggvenn (v 0.1.9)” (https://CRAN.R-project.org/package=ggvenn) was used to draw a Venn diagram to visualize the results.

### Identification of key genes

In order to investigate the protein-level interactions of candidate genes, we utilized the Search Tool for the Retrieval of Interacting Genes/Proteins (STRING) (https://www.string-db.org) to construct a PPI network (combined score ≥ 0.4). Subsequently, the PPI network was visualized by the Cytoscape package (v 3.10.2) [25]. Moreover, we employed the cytoHubba plugin in the Cytoscape package (v 3.10.2) to rank the candidate genes using 4 algorithms (Degree, Edge Percolated Component (EPC), Maximum Neighborhood Component (MNC), and Density of Maximum Neighborhood Component (DMN)). Then, the ggvenn package (v 0.1.10) was used to obtain the intersection of the top 10 genes selected by each of the 4 algorithms to acquire the candidate key genes. After that, the Wilcoxon test was used to contrast the expression abundances of candidate key genes among the IS group and the control group in the GSE58720 and GSE202659 datasets. Genes that had significantly different expression levels (*p* < 0.05) and consistent trends in both datasets were defined as key genes.

### Gene set enrichment analysis (GSEA) and Gene set variation analysis (GSVA)

The GSEA was conducted to elucidate the biological functions of key genes throughout the progression of IS. Initially, the Spearman correlation coefficients among each key gene and all other genes across all samples in the GSE58720 dataset were calculated using the psych package (v 2.4.3) (https://CRAN.R-project.org/package=psych), with the results then ordered in descending order. Subsequently, “c2.kegg.v7.5.1.entrez.gmt” was downloaded as a reference gene set from the Molecular Signatures Database (MSigDB) (https://www.gsea-msigdb.org/gsea/msigdb/index.jsp) using the msigdbr package (v 7.5.1) [75]. Following this, the GSEA was performed with the clusterProfiler package (v 4.8.3), with a threshold of |normalized enrichment score (NES)|> 1, False discovery rate (FDR) < 0.25, and *p* < 0.05. The GSVA was performed to elucidate the enriched pathway differences between disease and controls. First, the “h.hallmark.v7.4.symbols.gmt” from the Molecular Signatures Database (MSigDB) (https://www.gsea-msigdb.org/) was utilized as the reference gene set. Subsequently, GSVA was carried out with the GSVA package (v 1.42.0) with a threshold of |t|> 2 and *p* < 0.05[76].

### Construction of regulatory networks and GeneMANIA analysis

The regulatory mechanisms of key genes can also be probed based on the molecular level. The Transcriptional Regulatory Relationships Unraveled by Sentence-based Text mining (TRRUST) database (https://www.grnpedia.org/trrust/) was utilized to predict the transcription factors (TFs) that regulate key genes. In addition, the miRWalk database (http://mirwalk.umm.uni-heidelberg.de) was employed to forecast the miRNAs of key genes (the top 20 miRNAs with the smallest binding *p* values). Subsequently, the Cytoscape package (v 3.10.2) was adopted to construct the TFs-mRNAs network and the TFs-key genes-miRNAs regulatory network. After that, to reveal the relationships among key genes and predict the functions they participate in, based on the GeneMANIA database (https://genemania.org/), a co-expression network of key genes was developed to explore genes functionally related to key genes and their modes of functional interaction.

### Drug prediction and molecular docking

Drugs targeting key genes were predicted through the DrugBank database (https://go.drugbank.com/). The molecular formulas and 2 dimensional (2D) structures of the drugs were retrieved from the PubChem (https://pubchem.ncbi.nlm.nih.gov). The drug-key gene network was presented via the Cytoscape package (v 3.10.2). Among the predicted drugs, the one that targets the largest number of key genes and has the highest interaction score was identified as the key drug. Subsequently, convert the 2D structure into a 3D structure in Chem3D package (v 22.0.0) [77]. Finally, after hydrogenation in Autodock (http://autodock.scripps.edu/), it was saved in PDBQT format. In addition, the 3D configuration of the target protein of the key gene was retrieved using the IBDB database (https://www.rcsb.org/). Subsequently, molecular docking was carried out using the Autodock (http://autodock.scripps.edu/), and the results of molecular docking were visualized using PyMOL software (v 3.0.3) [78]. A molecular binding energy of ≤− 5.0 kcal/mol was considered to indicate good binding activity.

#### tMCAO

tMCAO procedure was performed in mice using an established method [79]. Briefly, anesthesia was induced and maintained with 1.5% isoflurane (RWD, Shenzhen, China), with body temperature kept normothermic throughout surgery. Following surgical exposure of the common carotid artery (CCA), external carotid artery (ECA), and internal carotid artery (ICA), a silicone-coated 6–0 monofilament (Covidien, St. Louis, MO) was inserted into the ECA. This filament was then advanced retrogradely to block the origin of the middle cerebral artery (MCA) for a duration of 90 min. Reperfusion commenced upon removal of the filament. Cerebral blood flow (CBF) was continuously assessed using laser Doppler flowmetry (Moor Instruments, Devon, UK). Occlusion was deemed successful if CBF decreased to ≤ 20% of pre-occlusion baseline levels, and reperfusion was confirmed by a return of CBF to ≥ 80% of baseline.

#### qRT-PCR

For the study, tissue samples from tMCAO (n = 3) and normal samples (n = 3) were collected from Fudan University and were approved by the Institutional Animal Care and Use Committee of Fudan University. Total RNA was extracted from tissues using TRI Reagent (Sigma-Aldrich, St. Louis, MO) via phenol–chloroform phase separation. Reverse transcription was performed using the PrimeScript RT Master Mix (Takara Bio, Dalian, China). PCR amplification was conducted on a StepOnePlus Real-Time PCR System (Thermo Fisher Scientific, Waltham, MA) under the following conditions: initial denaturation at 95 °C for 3 min, followed by 40 cycles of denaturation at 95 °C for 15 s and annealing/extension at 60 °C for 30 s. Gene expression levels were normalized to GAPDH, and primer sequences are listed in Additional file 4.

Reverse transcription was carried out with the TaqMan MicroRNA Reverse Transcription Kit (Applied Biosystems, Foster City, CA), followed by TaqMan probe-based quantification on a LightCycler 480 II platform (Roche, Basel, Switzerland). For every biological sample, 3 technical replicates were carried out. In the meantime, our study had been sanctioned via the Ethics Committee of Fudan University. The resulting data underwent statistical analysis and were depicted via Graphpad Prism (v 10.0) [80].

#### Statistical analysis

The statistical analyses were carried out with R software (v 4.3.1), and the notable group disparities were assessed by means of the Wilcoxon test. In qRT-PCR, the Ct values were compared using paired, two-tailed t-tests, which were performed using GraphPad Prism. A *p *value < 0.05 was considered statistically significant.

## Supplementary Information

Below is the link to the electronic supplementary material.

Additional file 1. Result of gene ontology (GO) enrichment analysis.

Additional file 2. Result of Kyoto encyclopedia of genes and genomes (KEGG) enrichment analysis.

Additional file 3. Association analysis of candidate genes with age **A** Intersection results of candidate genes with age-related neurological disease risk genes, **B**–**J** Single-cell expression profiles of 9 candidate genes **B** EMP3, **C** CD14, **D** CTSB, **E** MSR1, **F** SPP1, **G** TUBA1C, **H** RCAN1, **I** LGALS1, **J** LGALS3.

Additional file 4. qPR-PCR primer sequence.

## Data Availability

The datasets generated and/or analysed during the current study are available in the [GEO] repository, (http://www.ncbi.nlm.nih.gov/geo/). No datasets were generated or analysed during the current study.

## Abbreviations

AD: Alzheimer’s disease
IS: Ischemic stroke
MGGs: Microglia-related genes
DEGs: Differentially expressed genes
PPI: Protein-protein interaction
qRT-PCR: Quantitative real-time PCR
tMCAO: Transient middle cerebral artery occlusion
scRNA-seq: Single-cell RNA sequencing
hdWGCNA: High-dimensional weighted gene co-expression network analysis
WGS: Whole-genome sequencing
GEO: Gene expression omnibus
QC: Quality control
HVGs: Highly variable genes
PCA: Principal component analysis
UMAP: Uniform manifold approximation and projection
GO: Gene ontology
KEGG: Kyoto encyclopedia of genes and genomes
GSEA: Gene set enrichment analysis
GSVA: Gene set variation analysis
TFs: Transcription factors
miRNAs: MicroRNAs
CNS: Central nervous system
BBB: Blood-brain barrier
CCA: Common carotid artery
ECA: External carotid artery
ICA: Internal carotid artery
CBF: Cerebral blood flow
MCA: Middle cerebral artery
TLR: Toll-like receptor
NF-κB: Nuclear factor kappa-light-chain-enhancer of activated B cells
TNF-α: Tumor necrosis factor alpha
IL-1β: Interleukin-1 Beta
VEGF: Vascular endothelial growth factor
