# Supplementary figures and images for: Identification and validation of microglia-associated genes in ischemic stroke using single-cell and bulk RNA-seq
Source: Mol Brain. 2025 Dec 7;18:91. doi: 10.1186/s13041-025-01259-x (PMC12699842; doi:10.1186/s13041-025-01259-x)

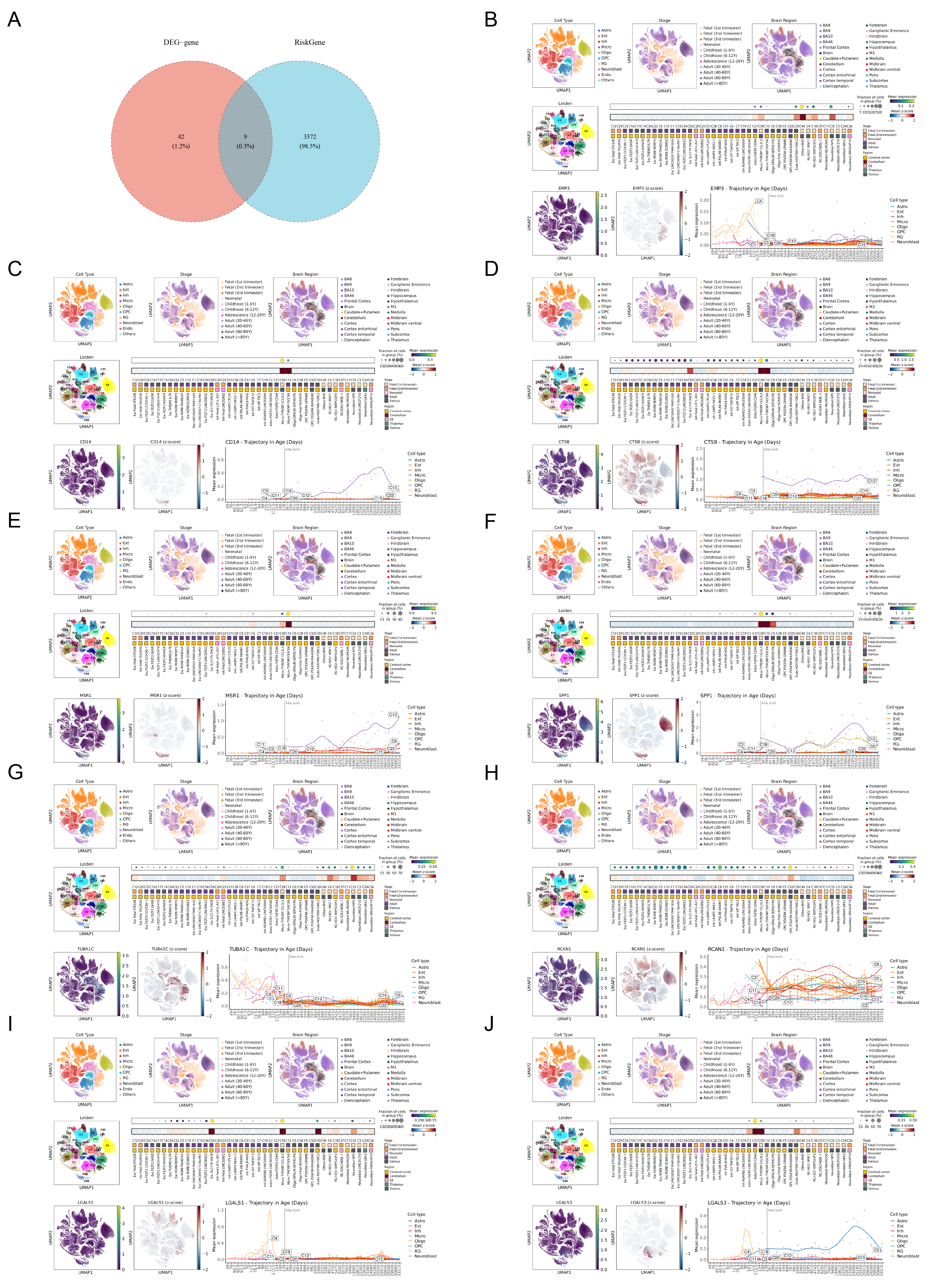

Supplement: Supplementary file 3 — Additional file 3. Association analysis of candidate genes with age A Intersection results of candidate genes with age-related neurological disease risk genes, B–J Single-cell expression profiles of 9 candidate genes B EMP3, C CD14, D CTSB, E MSR1, F SPP1, G TUBA1C, H RCAN1, I LGALS1, J LGALS3. [file 13041_2025_1259_MOESM3_ESM.tif]
