# Supplementary material for: Identification and validation of microglia-associated genes in ischemic stroke using single-cell and bulk RNA-seq
Source: Mol Brain. 2025 Dec 7;18:91. doi: 10.1186/s13041-025-01259-x (PMC12699842; doi:10.1186/s13041-025-01259-x)
Supplement: Supplementary file 4 — Additional file 4. qPR-PCR primer sequence. [file 13041_2025_1259_MOESM4_ESM.docx]

**Table S3 qPR-PCR primer sequence**

| Gene | Forward (5'-3') | Reverse (3'-5') |
| --- | --- | --- |
| Cd14 | TCTACCGACCATGGAGCGT | CCGCCGTACAATTCCACA |
| Csf1 | TGCTAAGTGCTCTAGCCGAG | CCCCCAACAGTCAGCAAGAC |
| Tlr2 | TTCAACAAGATCACCTACATTGGC | GGCGTCTCCCTCTATTGTATTGAT |
